# Development and Evaluation of Six Novel Recombinant GRA Proteins in Serodiagnosis of Human Toxoplasmosis

**DOI:** 10.3390/cimb47110879

**Published:** 2025-10-23

**Authors:** Karolina Sołowińska, Lucyna Holec-Gąsior

**Affiliations:** Department of Biotechnology and Microbiology, Faculty of Chemistry, Gdańsk University of Technology, 11/12 Narutowicza Str., 80-233 Gdańsk, Poland; karolina.solowinska@pg.edu.pl

**Keywords:** *Toxoplasma gondii*, toxoplasmosis, diagnosis, recombinant antigen, dense granule protein

## Abstract

*Toxoplasma gondii* is a globally distributed protozoan parasite, and reliable serodiagnosis is essential for effective management of toxoplasmosis. Conventional assays rely on tachyzoite lysate antigen (TLA), which suffers from limited standardization and reproducibility. In this study, immunodominant fragments of six dense granule proteins—GRA29, GRA35, GRA36, GRA45, GRA54, and GRA64—were expressed in *Escherichia coli*, purified, and evaluated as candidate antigens in IgG ELISAs using human sera. This study represents the first assessment of their diagnostic utility. Initial screening identified GRA29, GRA45, and GRA54 as promising candidates, with AUC values of 0.9983, 0.8507, and 0.9323, respectively, while GRA35-, GRA36-, and GRA64-based ELISA showed poor discrimination between seropositive and seronegative samples. Extended evaluation of GRA29-based assay with a larger serum panel (*n* = 286) confirmed excellent diagnostic performance, yielding an AUC of 0.9942 and higher sensitivity, specificity, positive predictive value (PPV), and negative predictive value (NPV) compared with TLA-ELISA. A comparative analysis revealed that GRA29 produced stronger reactivity in positive sera and lower background in negatives. These findings highlight GRA29 as a promising recombinant antigen for the serodiagnosis of human toxoplasmosis and a potential standardized alternative to TLA.

## 1. Introduction

*Toxoplasma gondii* is an obligate intracellular protozoan parasite and the causative agent of toxoplasmosis. Its broad host range, together with multiple routes of transmission, contributes to its global distribution in both humans and animals [1]. Members of the family *Felidae* serve as definitive hosts, while all other warm-blooded animals act as intermediate hosts [2]. Infection can occur via ingestion of sporulated oocysts from contaminated soil, water, or food, through consumption of undercooked meat containing tissue cysts, ingestion of unpasteurized milk or by vertical transplacental transmission; less commonly, it may follow blood transfusion or organ transplantation [3].

While *T. gondii* infection is prevalent, most immunocompetent individuals remain asymptomatic or develop only mild, self-limiting, flu-like symptoms. In contrast, toxoplasmosis represents a major health risk for immunocompromised patients, neonates, and congenitally infected fetuses. In individuals with impaired immunity, such as those with HIV/AIDS or undergoing immunosuppressive therapy, latent infections may reactivate and cause life-threatening complications [4]. Congenital toxoplasmosis occurs when primary infection is acquired during pregnancy, with severity influenced by gestational age and parasite strain virulence [5], and may lead to severe outcomes such as miscarriage, hydrocephalus, intracranial calcifications, chorioretinitis, or long-term neurological and ocular sequelae [6,7]. Beyond these established risks, an increasing body of evidence also links chronic infection to psychiatric and behavioral changes in humans, suggesting that its clinical impact may be broader than previously recognized [4].

Given the wide spectrum of clinical manifestations and the potential for severe outcomes, accurate diagnosis of toxoplasmosis is critical for disease management and prevention of complications. Laboratory diagnosis relies on detection of parasite-specific antibodies or DNA [8]. Molecular methods are mainly used in immunocompromised patients [9] and in prenatal cases, where PCR on amniotic fluid can confirm fetal infection [10]. In contrast, serological assays remain the routine diagnostic approach worldwide [11].

Despite their widespread use, serological assays face limitations in standardization and reproducibility, largely due to their reliance on tachyzoite lysate antigen (TLA), a protein mixture prone to batch variability. This heterogenicity affects sensitivity, specificity, and comparability between laboratories. Additional challenges include antibody cross-reactivity and differences in protocols and interpretation, which contribute to inconsistent results [8,12]. These drawbacks highlight the need for diagnostic tools that are reliable, standardized, and able to discriminate between infection stages. Recombinant antigens offer a promising alternative, providing consistent production, improved biosafety, and the ability to target immunodominant epitopes for enhanced accuracy [13].

Among the recombinant candidates under investigation, excretory–secretory antigens (ESA) have long been recognized for their strong immunogenicity and central role in host–parasite interactions [14,15]. Within this group, dense granule proteins (GRAs) are attractive as diagnostic candidates, as they are secreted in abundance during infection and serve as major components of both tachyzoite vacuoles and bradyzoite cyst walls [16]. Following invasion, the parasite establishes a parasitophorous vacuole (PV) that separates it from the host cytoplasm, and GRAs are secreted to remodel both the vacuolar lumen and the parasitophorous vacuole membrane (PVM). Within the vacuole, GRAs contribute to the formation of specialized structures, including the intravacuolar network (IVN) that facilitates nutrient acquisition and communication, and, during bradyzoite differentiation, the cyst wall that ensures parasite persistence during chronic infection. Other GRAs integrate into the PVM or are exported into the host cytoplasm and nucleus, where they modulate signaling and transcriptional pathways to subvert immune responses [17,18]. Collectively, these functions support parasite survival, replication, and dissemination, while simultaneously bringing GRAs into frequent contact with host defenses. Over the years, several GRA antigens have been explored both as tools to improve the serodiagnosis of *T. gondii* infection [11] and as promising targets for vaccine development [19,20].

This study set out to evaluate six dense granule proteins (GRA29, GRA35, GRA36, GRA45, GRA54, and GRA64) as recombinant antigens for the serodiagnosis of toxoplasmosis. To our knowledge, this represents the first assessment of their diagnostic utility. These proteins were expressed in *Escherichia coli* and their immunoreactivity with human sera was assessed using enzyme-linked immunosorbent assays (ELISAs). Our results highlight GRA29 as a promising recombinant antigen with superior diagnostic performance compared to TLA.

## 2. Materials and Methods

### 2.1. Serum Samples

Human serum samples were obtained from the collection of the Department of Biotechnology and Microbiology, Gdańsk University of Technology, Poland, and originated from routine toxoplasmosis screening performed in Poland. All sera were anonymized prior to analysis, and no identifying information about the donors was available to the researchers. All procedures performed in this study were conducted in accordance with the ethical principles of the 1964 Declaration of Helsinki and its subsequent amendments, or comparable ethical standards.

Samples were categorized as either seropositive or seronegative for *T. gondii* on the basis of results obtained with commercial assays: VIDAS TOXO IgM, VIDAS TOXO IgG II, and VIDAS TOXO IgG Avidity tests (bioMérieux, Marcy l’Etoile, France). These assays were used to determine the presence of specific anti-*T. gondii* IgM and IgG antibodies and to evaluate IgG avidity, allowing classification of sera into negative, acute, or chronic infection groups.

For the preliminary evaluation of antigen immunoreactivity, ELISA assays based on each antigen were performed with the same 24 seropositive (IgG^+^, IgM^+^/^−^) and 24 seronegative (IgG^−^, IgM^−^) serum samples.

For the extended analysis, a total of 286 human serum samples were analyzed by ELISA, and each assessed antigen was tested using this same panel of sera. These included 136 seronegative samples (IgG^−^, IgM^−^), 150 seropositive sera comprising 33 samples from patients with acute infection (IgG^+^, IgM^+^/^−^, low IgG avidity), and 117 samples from individuals with chronic infection (IgG^+^, IgM^−^, high IgG avidity). The chronic group was further subdivided according to IgG concentration: >300 IU/mL, 100–300 IU/mL, and <100 IU/mL [21].

### 2.2. Construction of Recombinant Plasmids

Coding sequences corresponding to selected antigenic regions of GRA29_425–865_ (GenBank: EPR64817.1), GRA35_142–378_ (GenBank: EPR62264.1), GRA36_210–452_ (GenBank: EPR59512.1), GRA45_33–394_ (GenBank: EPR58836.1), GRA54_501–800_ (GenBank: EPR61484.1), and GRA64_20–263_ (GenBank: EPT30204.1) were amplified by PCR using *T. gondii* (RH strain) cDNA as the template. Amplification reactions were carried out with the CloneAmp HiFi PCR Premix (Takara Bio USA, Inc., San Jose, CA, USA) according to the manufacturer’s instructions. The specific primer pairs used are listed in Table 1.

Purified PCR products were subsequently inserted into a pUET1 vector [22] using the In-Fusion^®^ HD Cloning Kit (Takara Bio USA, Inc., San Jose, CA, USA). The expression vector was linearized by PCR with primers pUET1.FOR (5′-acccagatctgggctgtcc-3′) and pUET1.REV (5′-aattcgagctccgtcgacaag-3′). All constructs were designed in-frame with the N- and C-terminal His_6_-tag coding regions, enabling affinity purification. Correct insertion of the DNA fragments was confirmed by DNA sequencing (Genomed S.A., Warsaw, Poland).

### 2.3. Expression and Purification of Recombinant Proteins

Recombinant plasmids pUET1/GRA29, pUET1/GRA35, pUET1/GRA36, pUET1/GRA45, pUET1/GRA54, and pUET1/GRA64 were transformed into *E. coli* strains BL21(DE3)pLysS, BL21(DE3)placI, and Rosetta(DE3)pLysS. Overnight cultures were inoculated into LB broth supplemented with 100 μg/mL ampicillin and 34 μg/mL chloramphenicol and grown with shaking to an OD_600_ of 0.4. Protein expression was induced with isopropyl β-D-1-thiogalactopyranoside (IPTG) at a final concentration of 1 mM, and cells were further incubated with vigorous shaking for up to 18 h. Culture conditions, including the expression strain, post-induction temperature, and post-induction incubation time, were optimized individually for each recombinant protein.

The expected molecular weights and theoretical isoelectric points (pI) of each protein were calculated using the Compute pI/Mw tool available at ExPASy (https://web.expasy.org/compute_pi, accessed on 19 October 2025).

Cells were harvested by centrifugation and disrupted by sonication. Following clarification by centrifugation, recombinant proteins were purified from the supernatant in a single-step immobilized metal affinity chromatography (IMAC) procedure using Ni^2+^-IDA Sepharose resin (Novagen, Madison, WI, USA) according to the manufacturer’s instructions. The obtained protein preparations were analyzed on 12% SDS–PAGE gels stained with Coomassie Brilliant Blue. Protein concentrations were determined by the Bradford assay with bovine serum albumin (BSA) as a standard (Bio-Rad, Hercules, CA, USA).

### 2.4. Preparation of T. gondii Tachyzoite Lysate Antigen (TLA)

*Toxoplasma* lysate antigen was prepared as described previously [23]. Parasites were resuspended in distilled water and subjected to repeated freeze–thaw cycles. The suspension was clarified by sequential centrifugation, and the resulting supernatant was stored at −70 °C until use. Protein concentration in the TLA preparation was determined using the Bradford assay with BSA as a reference standard (Bio-Rad, Hercules, CA, USA).

### 2.5. IgG ELISA

Ninety six well microtiter plates (MaxiSorp, Thermo Fisher Scientific, Inc., Waltham, MA, USA) were coated overnight at 4 °C with either 2.5 µg/mL of recombinant GRAs or 1 µg/mL of TLA diluted in 0.05 M carbonate–bicarbonate buffer (pH 9.6). Plates were washed three times (50 mM Tris; 0.88% NaCl; 0.1% Tween 20, pH 7.4) and blocked with 1% (*w*/*v*) BSA in phosphate-buffered saline containing 0.05% Tween-20 (PBST) for 1 h at 37 °C. After another three washes, human serum samples were applied at a dilution of 1:100 in blocking buffer and incubated for 1 h at 37 °C. After washing, bound IgG was detected with HRP-conjugated goat anti-human IgG antibodies (1:16,000; Jackson ImmunoResearch, Ely, UK), followed by incubation for 1 h at 37 °C. Colour was developed using o-phenylenediamine dihydrochloride (OPD) substrate (SIGMAFAST™ OPD, Sigma, Saint Louis, MO, USA) for 30 min in the dark at 37 °C, and the reaction was stopped with 2 M H_2_SO_4_. Absorbance was measured at 492 nm using a Multiskan FC microplate reader (Thermo Fisher Scientific, Inc., Waltham, MA, USA). Each serum sample was tested in duplicate wells, and the mean absorbance value was used for analysis.

To exclude the possibility of background signal or cross-reactivity with bacterial components, an additional ELISA was performed using purified *E. coli* lysate as the coating antigen. All sera tested yielded low reactivity, with a mean absorbance value of 0.164 ± 0.044.

### 2.6. Statistical Analysis

All statistical analyses were conducted using GraphPad Prism version 10.6.0 (GraphPad Software, Boston, MA, USA).

For the preliminary evaluation, OD_492_ values obtained for ELISAs based on each recombinant antigen and TLA were first tested for normality using the Shapiro–Wilk test. As distributions did not meet assumptions of normality across all groups, comparisons of OD values between seropositive and seronegative sera were performed using the non-parametric Mann–Whitney U test. Diagnostic performance of each antigen was assessed by receiver operating characteristic (ROC) curve analysis. The area under the curve (AUC) with 95% confidence intervals (CI) was calculated, and optimal cut-off values were determined using the Euclidean distance criterion [24]. At these thresholds, sensitivity and specificity (with 95% CI) were established, and Fisher’s exact test was applied to evaluate the association between ELISA results and the reference classification.

For the extended evaluation of TLA- and GRA29-based ELISAs, ROC analyses were repeated, with AUC, sensitivity, specificity, positive predictive value (PPV) and negative predictive value (NPV) calculated, all with 95% CI. At the optimal cut-offs, Fisher’s exact test was again used to test the association between ELISA classifications and the reference standard. Paired OD values obtained with GRA29- and TLA-coated plates on the same serum samples were also compared. Normality of paired differences was tested with the Shapiro–Wilk test, and as the data deviated from normality, the Wilcoxon signed-rank test was applied. A *p* value < 0.05 was considered statistically significant.

## 3. Results

### 3.1. Construction of Recombinant Plasmids, Expression and Purification of Recombinant Proteins

The recombinant plasmids (pUET1/GRA29, pUET1/GRA35, pUET1/GRA36, pUET1/GRA45, pUET1/GRA54, and pUET1/GRA64) were successfully constructed. Plasmid maps are shown in Figures S1–S6 (Supplementary Materials).

All six recombinant GRA proteins were successfully expressed in *E. coli* following IPTG induction, with optimal expression conditions for each summarized in Table 2. The expected molecular weights and theoretical pI of each protein are summarized in Table 3.

Recombinant proteins were purified, resulting in single major bands corresponding to the expected molecular weights, at sufficient electrophoretic purity for immunoassay application, as confirmed by SDS–PAGE analysis (shown in Figure S7 of Supplementary Materials). Yields per liter of bacterial culture are presented in Table 4.

### 3.2. IgG ELISA

#### 3.2.1. Preliminary Evaluation of Diagnostic Performance

**Figure 1:**
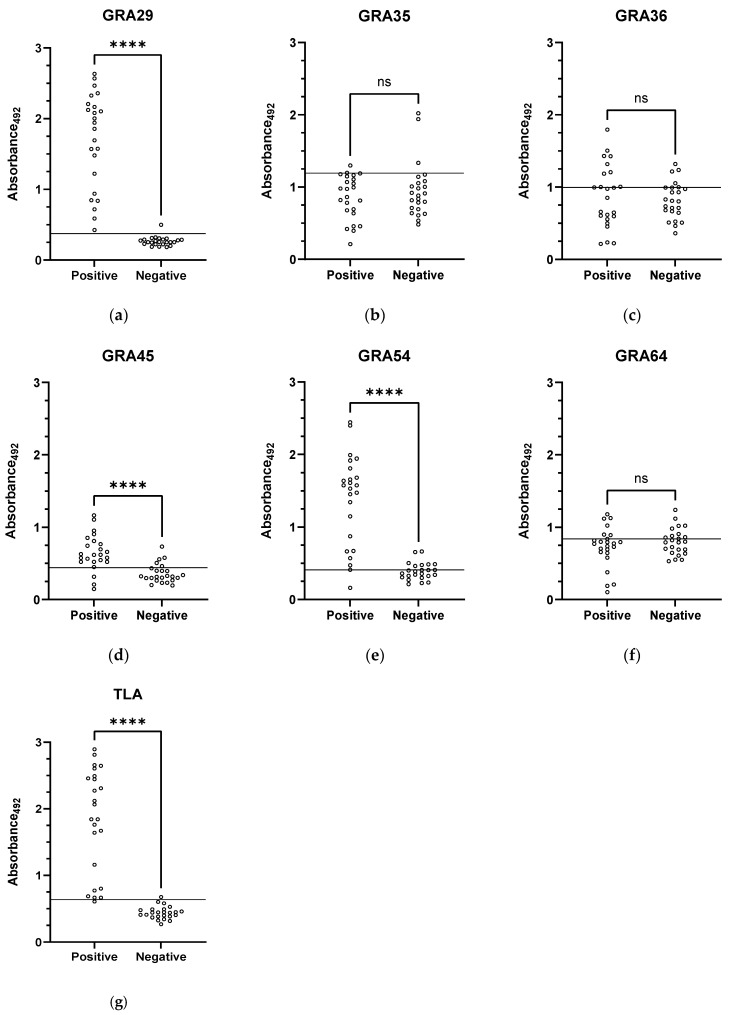
Scatter plots of ELISA absorbance values (OD_492_) for (**a**) GRA29-, (**b**) GRA35-, (**c**) GRA36-, (**d**) GRA45-, (**e**) GRA54-, (**f**) GRA64-, and (**g**) TLA-coated plates, obtained using seropositive (*n* = 24) and seronegative (*n* = 28) human serum samples. Horizontal lines indicate cut-off values determined by ROC curve analysis. Statistical comparisons were performed using the Mann–Whitney U test; ns, not significant; **** *p* < 0.0001.

To assess the diagnostic potential of the six recombinant GRA proteins, an initial ELISA screening was performed using a panel of 48 human serum samples (24 seropositive and 24 seronegative), with TLA included as a reference antigen. Scatter plot analysis of absorbance values illustrates the differences in diagnostic performance (Figure 1). GRA29-, GRA45-, and GRA54-ELISA produced a clear separation between seropositive and seronegative sera, with statistically significant differences in OD distributions (*p* < 0.0001). GRA35-, GRA36-, and GRA64-based immunoassays, however, showed substantial overlap between groups, with no significant differences detected (ns).

**Figure 2:**
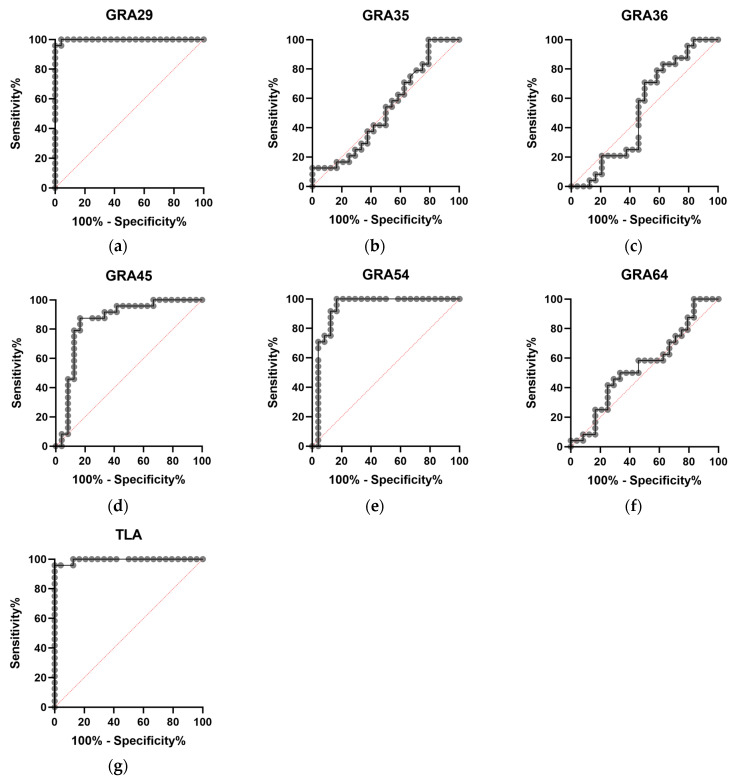
Receiver operating characteristic (ROC) curves of (**a**) GRA29-, (**b**) GRA35-, (**c**) GRA36-, (**d**) GRA45-, (**e**) GRA54-, (**f**) GRA64-, and (**g**) TLA-based IgG ELISAs. The diagonal red dotted line represents the line of no discrimination (AUC = 0.5).

ROC curve analysis (Figure 2) further revealed marked differences in diagnostic performance among the tested proteins. GRA29-, GRA45-, and GRA54-based assays showed strong discriminatory capacity between seropositive and seronegative sera, with AUC values of 0.9983, 0.8507, and 0.9323, respectively. Fisher’s exact test confirmed statistically significant associations with the reference classification (*p* < 0.0001). In contrast, GRA35-, GRA36-, and GRA64-ELISA displayed poor diagnostic performance, with AUC values ranging from 0.5260 to 0.5503 and no significant association with the reference classification (Fisher’s exact test, ns). A summary of AUC values, optimal cut-offs, sensitivities, specificities, and Fisher’s exact test results is provided in Table 5.

Although ELISAs based on GRA29, GRA45, and GRA54 demonstrated promising diagnostic capacity, only GRA29 was selected for extended evaluation. ELISA based on this antigen achieved near-perfect classification of sera, with an AUC, sensitivity, and specificity comparable to or exceeding that of TLA-based assay. GRA45-ELISA showed overall weaker diagnostic accuracy, whereas GRA54-ELISA displayed high AUC and sensitivity but reduced specificity, resulting in an increased rate of false positives relative to TLA-ELISA. Thus, while GRA45- and GRA54-based assays are diagnostically relevant, they do not provide a feasible alternative to TLA-based detection, in contrast to GRA29-ELISA, which showed superior overall performance.

#### 3.2.2. Extended Evaluation of GRA29-Based IgG ELISA

**Figure 3:**
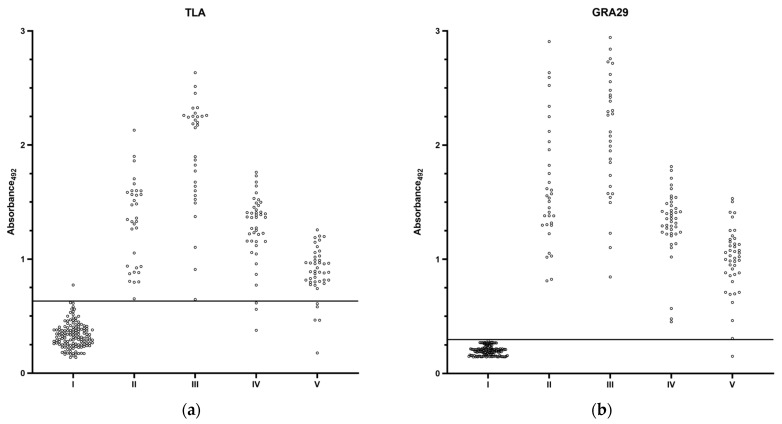
Scatter plots of ELISA absorbance values (OD_492_) for (**a**) TLA-coated plates; (**b**) and GRA29-coated plates across serum groups. I: seronegative (IgG^−^, IgM^−^; *n* = 136); II: acute infection (IgG^+^, IgM^+^/^−^, low IgG avidity; *n* = 33); III: chronic infection, high IgG (IgG^+^, IgM^−^, high IgG avidity, IgG > 300 IU/mL; *n* = 31); IV: chronic infection, medium IgG (IgG^+^, IgM^−^, high IgG avidity, IgG 100–300 IU/mL; *n* = 43); V: chronic infection, low IgG (IgG^+^, IgM^−^, high IgG avidity, IgG < 100 IU/mL; *n* = 43). The horizontal line represents the assay-specific cut-off determined by ROC analysis.

To fully determine the reactivity of the selected recombinant antigen, GRA29 and TLA were used in an indirect IgG ELISA on a total of 286 human serum samples categorized into the following five groups: I: seronegative; II: acute infection; III: chronic infection, high IgG; IV: chronic infection, medium IgG; V: chronic infection, low IgG. The distribution of OD values for the different serum groups is shown in Figure 3. The GRA29-based ELISA misclassified only sera from the low-IgG chronic group, whereas the TLA-based assay showed false negatives not only in this group but also among medium-IgG chronic samples. In addition, acute and high-IgG chronic samples produced OD values close to the cut-off in the TLA assay, while the GRA29 assay consistently yielded strong positive signals in these groups. Paired comparisons of OD values revealed that GRA29 produced significantly higher reactivity in positive sera (Wilcoxon signed-rank test, median difference = 0.1005, *p* < 0.0001) while maintaining significantly lower background reactivity in negative sera (median difference = −0.1090, *p* < 0.0001).

**Figure 4:**
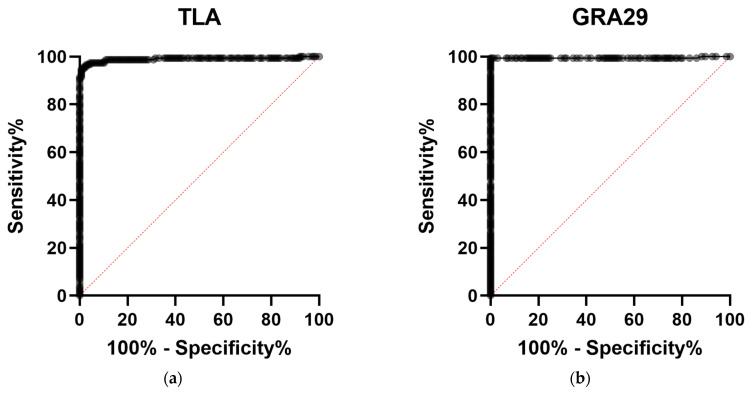
Receiver operating characteristic (ROC) curves of ELISA assays for detection of *T. gondii*-specific IgG. (**a**) TLA-based ELISA; (**b**) GRA29-based ELISA. The diagonal red dotted line indicates the line of no discrimination (AUC = 0.5).

ROC curve analysis demonstrated excellent diagnostic performance for both assays, with an AUC of 0.9894 (95% CI: 0.9765–1.000) for TLA-ELISA and 0.9942 (95% CI: 0.9828–1.000) for GRA29-ELISA (Figure 4, Table 6).

At the optimal cut-off values, GRA29-ELISA exhibited superior discrimination between seropositive and seronegative samples, with higher sensitivity, specificity, PPV, and NPV compared with TLA-based assay. These parameters are summarized in Table 6. Fisher’s exact test further demonstrated that classifications obtained with both TLA- and GRA29-based assays were significantly associated with the reference standard (*p* < 0.0001), further validating that both tests are diagnostically meaningful.

## 4. Discussion

Efforts to improve the serodiagnosis of toxoplasmosis have long focused on identifying recombinant antigens that could replace TLA, which provides broad antigenic coverage, however its heterogeneity poses challenges for assay reproducibility and standardization. Recombinant proteins offer a solution by enabling consistent production and targeted selection of specific epitopes and/or immunodominant fragments. Among these, dense granule proteins have been widely studied. Several GRAs, including GRA1 [25,26,27], GRA2 [27,28,29,30], GRA4 [27,31], GRA5 [23], GRA6 [25,32,33,34], GRA7 [27,28,31,34,35], GRA8 [27] and GRA14 [34] have demonstrated diagnostic potential for detecting anti-*T. gondii* IgG. Notably, several studies have shown that combinations of recombinant antigens achieve higher sensitivity and specificity than single antigens alone [23,25,26,27,28,31], likely due to broader epitope coverage, though at the expense of increased production complexity and cost. As an emerging alternative, chimeric antigens that combine immunodominant fragments of different proteins into a single fusion construct are being explored to enhance diagnostic accuracy while reducing assay complexity [36,37,38].

Despite these advances, no single recombinant antigen has yet replaced TLA in routine serological testing. Nevertheless, their established diagnostic potential suggests that unexplored members of this protein family may provide additional value. To address this, we selected six previously untested GRAs (GRA29, GRA35, GRA36, GRA45, GRA54, and GRA64) for evaluation of their utility as recombinant serodiagnostic antigens.

These dense granule proteins have varying roles in *Toxoplasma* biology. GRA29 is secreted into the PV, where it forms filamentous structures distinct from the intravacuolar network and previously described actin filaments, and it relocalizes during stage conversion to bradyzoites [39]. It has also been shown to be non-essential for parasite survival in vivo [40]. GRA35 has been linked to inflammasome activation. Together with GRA42 and GRA43, it functions as a key factor required for activation of the NLRP1 inflammasome and induction of pyroptosis in Lewis rat macrophages [41,42]. GRA35 has also been investigated for its protective potential in experimental vaccination studies in mouse models. Immunization with recombinant protein cocktails including GRA35 [43], as well as DNA vaccines encoding GRA35 in combination with GRA42 and GRA43 [44], elicited humoral and cellular immune responses. GRA36 is less well characterized; while it shares >40% sequence similarity with GRA35, gene knockout studies indicate that it does not share the same inflammasome-activating function [41].

Other candidates included GRA45, a chaperone-like protein essential for the correct localization of GRAs into the PVM and for the export of dense granule effectors into host cells [45,46,47]. Parasites lacking GRA45 show impaired survival under IFNγ-mediated pressure and reduced virulence in mice [47,48]. On the other hand, little is known about the precise biological role of GRA54, it has been identified as a MYR1-associated protein; however, disruption of GRA54 did not produce detectable effects on effector translocation across the PVM [45]. Finally, GRA64 is a transmembrane protein localized to the PVM and partly exposed to the host cytoplasm during tachyzoite and bradyzoite stages. Although implicated in vesicular trafficking, its disruption does not affect virulence or cyst formation in mice [49]. While genetic disruption of some of these proteins does not alter parasite virulence in mice, this does not exclude the possibility that they contribute to host immune recognition.

To the best of our knowledge, only GRA29 and GRA35 have previously been produced in recombinant form, and none of the six tested antigens have been assessed for diagnostic performance. Thus, establishing their expression in *E. coli*, purification, and evaluation in IgG-based ELISAs with human sera represents a novel contribution of this study.

The vast majority of studies evaluating recombinant *T. gondii* antigens have relied on prokaryotic expression systems [50]. In the present study, the bacterial expression strategy yielded protein preparations with production levels ranging from 18.1 to 164.8 mg/L of culture. Although few studies report detailed production efficiencies, the yields obtained here are comparable to, and in some cases exceed those previously described for recombinant GRA proteins. For instance, Golkar et al. reported a yield of 12 mg/L for GRA2 [30], while another study achieved 28 mg/L for the same antigen [29]. GRA5 and GRA8 have been produced with reported yields of 15 mg/L [23] and 68 mg/L [51], respectively. In comparison, the production levels obtained in the present study, particularly for GRA35, GRA36, and GRA45 (exceeding 160, 130, and 120 mg/L of bacterial culture, respectively), show the efficiency of the developed expression systems. Such high yields are advantageous not only for research purposes but also for the potential development of standardized diagnostic assays, where scalability and cost-effectiveness of antigen production are important.

The evaluation of six recombinant GRA proteins revealed marked differences in their diagnostic potential. Among the tested candidates, GRA29, GRA45, and GRA54 demonstrated the highest discriminatory ability, with AUC values ranging from 0.85 to 0.99 and highly significant associations with the reference classification. These findings were further supported by clear separation of seropositive and seronegative sera and statistically significant differences in their absorbance values. In contrast, GRA35, GRA36, and GRA64 showed poor performance, with AUC values close to random classification and no significant differences between groups, indicating limited applicability for serological detection of *T. gondii* infection.

Further inspection of the data underscores the unique diagnostic value of GRA29. This antigen combined high sensitivity (95.83%) with perfect specificity (100%), outperforming TLA. GRA45, while diagnostically relevant, displayed lower sensitivity (79.17%) and specificity (87.50%), while GRA54, despite achieving high specificity (95.83%) and a good AUC (0.9323), showed a higher rate of false-negative classifications compared with TLA. These limitations reduce their feasibility as standalone diagnostic tools. Therefore, GRA29 was chosen for extended analysis on a larger pool of human sera.

The extended comparison of TLA- and GRA29-IgG ELISAs shows that although both assays perform very well, the GRA29-based consistently outperforms the TLA-based ELISA, offering higher AUC values, sensitivity, specificity, PPV, and NPV at the optimal cut-off, stronger reactivity in positive sera, and lower background in negative sera. Moreover, scatter plot analyses confirmed that the GRA29-based ELISA provided more robust performance across serum groups, with fewer misclassifications and borderline results. Thus, GRA29 represents a promising recombinant antigen for the serodiagnosis of toxoplasmosis and may serve as a standardized alternative to TLA.

Previous studies assessing recombinant GRA antigens have generally shown that, while they can elicit strong antibody responses, their overall discriminatory power between seropositive and seronegative individuals remains limited. Lecordier et al. [25] reported that a GRA1-based IgG ELISA achieved 68% sensitivity, while Luo et al. [28] found that GRA2 reached 85% sensitivity and 85% specificity. In contrast, Aubert et al. [27] excluded GRA1, GRA2, and GRA4 at the preliminary stage of reactivity assessment due to insufficient performance. Consistent with this, Nigro et al. [31] reported low diagnostic accuracy for GRA4, with sensitivity of 58.3% in acute and only 18.2% in chronic infection. GRA5 [23] and GRA14 [34] have also been evaluated, with ELISAs based on these antigens correctly classifying 70.9% and 40.7% of seropositive samples, respectively. Among the GRAs tested, GRA7 has emerged as one of the most promising, with several studies reporting high sensitivity and specificity across infection stages. However, its diagnostic performance has not been consistent. Ybanez et al. [34] showed 100% sensitivity and specificity, while Arab-Mazar et al. [35] reported values of 92% and 94%, respectively. Aubert et al. [27] found that GRA7-based ELISA correctly classified 83.40% of seropositive samples, and Luo et al. [28] observed 83.3% sensitivity and 90% specificity. In contrast, Nigro et al. [31] reported more modest performance, with 75% sensitivity in acute and only 36.3% in chronic cases.

Many GRAs have demonstrated higher sensitivity in acute infection but markedly reduced reactivity in chronic cases, positioning them as potential markers of infection stage. For example, ELISAs based on GRA2 and GRA6 achieved sensitivities above 90% for acute sera, yet performance dropped substantially in chronic infection, in some cases below 40% [30,32,33,52]. Similarly, GRA8-based assay correctly classified 70.04% of all seropositive samples but showed only 37.14% sensitivity in chronic infection compared with 98.87% in acute cases [27].

In the presented findings GRA29 exhibited strong reactivity with IgG antibodies in both acute and chronic sera, resulting in near-perfect discrimination between seropositive and seronegative individuals. While this broad reactivity limits its ability to distinguish infection stage, it positions GRA29 as a robust antigen for universal serological screening, where it could be applied as a primary diagnostic marker.

This study has certain limitations that should be considered when interpreting the findings. The sample size of tested human sera, while adequate for preliminary evaluation, was relatively limited, and future studies should examine much larger cohorts to increase statistical power and improve the generalizability of the results. Furthermore, this work focused solely on the detection of anti-*T. gondii* IgG antibodies. Additional studies should assess the diagnostic potential of these antigens in IgM-based assays and in IgG avidity testing, which are particularly relevant for distinguishing acute from chronic infections. Future research could also investigate whether GRA29 reliably recognized anti-*T. gondii* IgG in other intermediate hosts, broadening its applicability to both clinical and veterinary settings. Addressing these limitations will fully establish the diagnostic value of the investigated proteins.

## 5. Conclusions

The objective of this study was to biotechnologically produce and assess the diagnostic performance of six recombinant dense granule proteins—GRA29, GRA35, GRA36, GRA45, GRA54, and GRA64—for the serological detection of *T. gondii* infection. All proteins were successfully expressed in *E. coli*, purified, and evaluated by IgG-based ELISA using human sera.

Among the tested candidates, GRA29, GRA45, and GRA54 exhibited the highest discriminatory power, with AUC values ranging from 0.8507 to 0.9983. These proteins showed strong correlations with reference classifications, and statistically significant differences in absorbance values between seropositive and seronegative sera confirmed their diagnostic relevance. In contrast, GRA35, GRA36, and GRA64 demonstrated low diagnostic utility, with AUC values approaching random classification and no significant separation between positive and negative samples.

Overall, the results indicate that while most of the tested recombinant GRA proteins show limited potential for routine diagnostic application, GRA29 in particular displays strong reactivity, supporting its potential as a standardized recombinant antigen for reliable serodiagnosis of human toxoplasmosis.

## Figures and Tables

**Table 1:** Primers for the amplification of genes coding the selected immunodominant protein fragments. Lowercase letters correspond to the nucleotides homologous to the linearized vector, whereas uppercase letters are nucleotides homologous to the target gene.

Primer Name	Primer Sequence	Amplicon Size [bp]
gra29FOR	agcccagatctgggtCAAATCTTGGCTCTGCTTC	1354
gra29REV	gacggagctcgaattACGTGTCCCTCTTCCC	
gra35FOR	agcccagatctgggtGCGAAGCGTCAATATGAG	742
gra35REV	gacggagctcgaattAGTCTGTTTCGGCTCCG	
gra36FOR	agcccagatctgggtTATCGGCGCTATCGTGAT	760
gra36REV	gacggagctcgaattCGTACGCTGTGCCC	
gra45FOR	gacggagctcgaattCGTACGCTGTGCCC	1117
gra45REV	gacggagctcgaattCGTACGCTGTGCCC	
gra54FOR	agcccagatctgggtGGTGCAACACGAGGAC	931
gra54REV	gacggagctcgaattTTGTGCCCTCACACCAG	
gra64FOR	gacggagctcgaattCGTCTTAAACCCCTTCAAGC	763
gra64REV	agcccagatctgggtGCAAACTACGATTTCTTTGTTCTT	

**Table 2:** Summary of optimal expression conditions for GRA29, GRA35, GRA36, GRA45, GRA54, and GRA64, including *E. coli* expression strain, post-induction temperature and post-induction incubation time.

Protein Name	*E. coli* Expression Strain	Post-Induction Temperature [°C]	Post-Induction Incubation Time [h]
GRA29	BL21(DE3)pLacI	37	18
GRA35	BL21(DE3)pLacI	25	18
GRA36	Rosetta(DE3)pLysS	25	18
GRA45	BL21(DE3)pLysS	30	6
GRA54	BL21(DE3)pLacI	25	18
GRA64	BL21(DE3)pLacI	37	18

**Table 3:** Predicted molecular weights and theoretical isoelectric points (pI) of recombinant GRA29, GRA35, GRA36, GRA45, GRA54, and GRA64, calculated from their amino acid sequences using the ExPASy Compute pI/Mw tool.

Protein Name	Molecular Weight [kDa]	Isoelectric Point
GRA29	52.0	8.03
GRA35	33.7	6.14
GRA36	34.5	6.43
GRA45	47.7	5.17
GRA54	40.1	9.64
GRA64	32.5	7.95

**Table 4:** Protein yields obtained per liter of bacterial culture.

Protein Name	Yield [mg]
GRA29	28.3
GRA35	164.8
GRA36	138.0
GRA45	123.0
GRA54	18.1
GRA64	38.0

**Table 5:** Diagnostic performance of recombinant GRA proteins and TLA in ELISA-based detection of *T. gondii*-specific IgG antibodies.

Antigen	AUC ^1^ (95% CI)	Cut-Off	Sensitivity [%] (95% CI)	Specificity [%] (95% CI)	Fisher’s Exact *p*-Value
GRA29	0.9983 (0.9925–1.000)	0.3740	95.83 (79.76–99.79)	100.00 (86.20–100.00)	<0.0001
GRA35	0.5269 (0.3600–0.6938)	1.193	12.50 (4.34–31.00)	91.67 (74.15–98.52)	ns ^2^
GRA36	0.5260 (0.3544–0.6977)	0.9945	79.17 (59.53–90.76)	41.67 (24.47–61.17)	ns
GRA45	0.8507 (0.3544–0.6977)	0.4415	79.17 (59.53–90.76)	87.50 (69.00–95.66)	<0.0001
GRA54	0.9323 (0.8464–1.000)	0.4090	70.83 (50.83–85.09)	95.83 (79.76–99.79)	<0.0001
GRA64	0.5503 (0.3848–0.7159)	0.8390	41.67 (24.47–61.17)	75.00 (55.10–88.00)	ns
TLA	0.9948 (0.9822–1.000)	0.6355	95.83 (79.76–99.79)	95.83 (79.76–99.79)	<0.0001

^1^ AUC—area under the curve; ^2^ ns—not significant.

**Table 6:** Diagnostic performance of TLA- and GRA29-based IgG ELISAs.

Antigen	AUC ^1^ (95% CI)	Cut-Off	Sensitivity [%] (95% CI)	Specificity [%] (95% CI)	PPV ^2^ [%]	NPV ^3^ [%]
TLA	0.9894 (0.9765–1.000)	0.6320	99.30 (96.15–99.96)	94.41 (89.35–97.14)	94.67 (89.83–97.27)	99.26 (95.95–99.96)
GRA29	0.9942 (0.9828–1.000)	0.2965	100.00 (97.49–100.00)	99.27 (95.98–99.96)	99.33 (96.32–99.97)	100.00 (97.25–100.00)

^1^ AUC—area under the curve; ^2^ PPV—positive predictive value; ^3^ NPV—negative predictive value.

## Data Availability

The original contributions presented in this study are included in the article/Supplementary Materials. Further inquiries can be directed to the corresponding author.

## Supplementary Materials

The following supporting information can be downloaded at https://www.mdpi.com/article/10.3390/cimb47110879/s1.

## Abbreviations

The following abbreviations are used in this manuscript:

TLA	Tachyzoite lysate antigen
ESA	Excretory–secretory antigens
GRA	Dense granule proteins
PV	Parasitophorous vacuole
PVM	Parasitophorous vacuole membrane
IVN	Intravacuolar network
ELISA	Enzyme-linked immunosorbent assay
OD	Optical density
PPV	Positive predictive value
NPV	Negative predictive value
AUC	Area under the curve
ROC	Receiver operating characteristic
