# Supplementary material for: Development and Evaluation of Six Novel Recombinant GRA Proteins in Serodiagnosis of Human Toxoplasmosis
Source: Curr Issues Mol Biol. 2025 Oct 23;47(11):879. doi: 10.3390/cimb47110879 (PMC12651256; doi:10.3390/cimb47110879)
Supplement: Supplementary file 1 [file cimb-47-00879-s001.zip › cimb-3934191-supplementary.pdf]

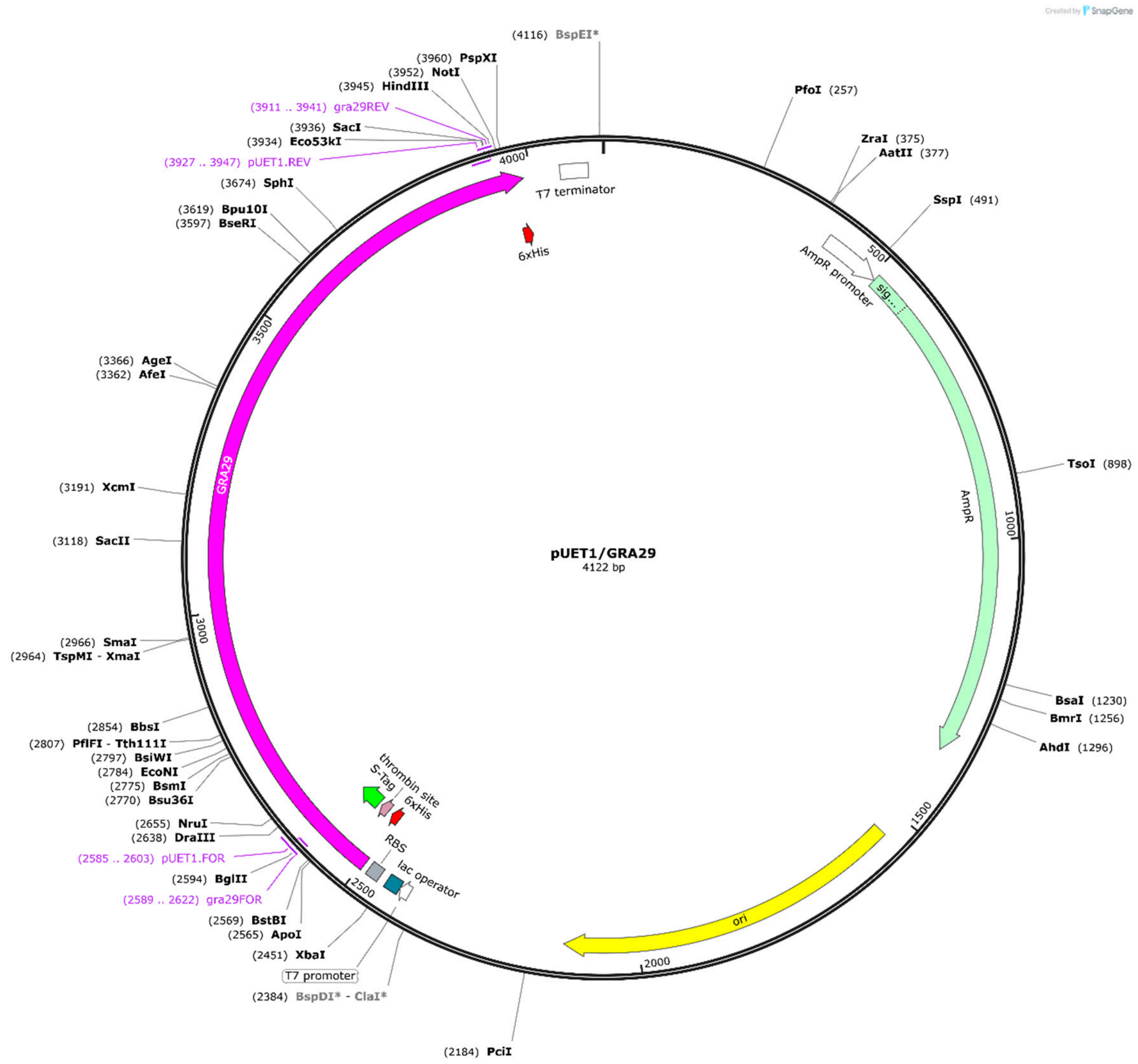

Figure S1. pUET1/GRA29 map.

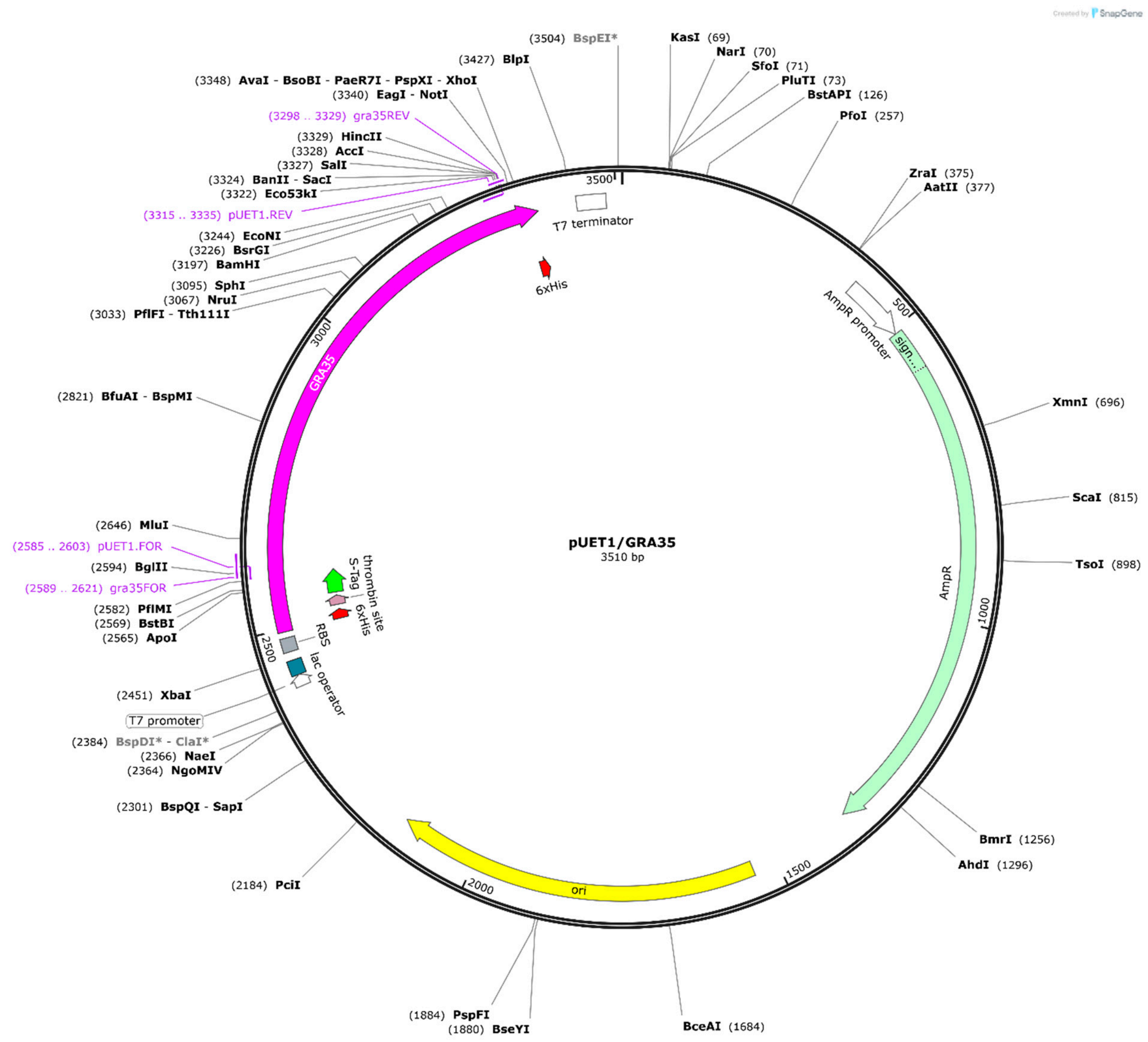

Figure S2. pUET1/GRA35 map.

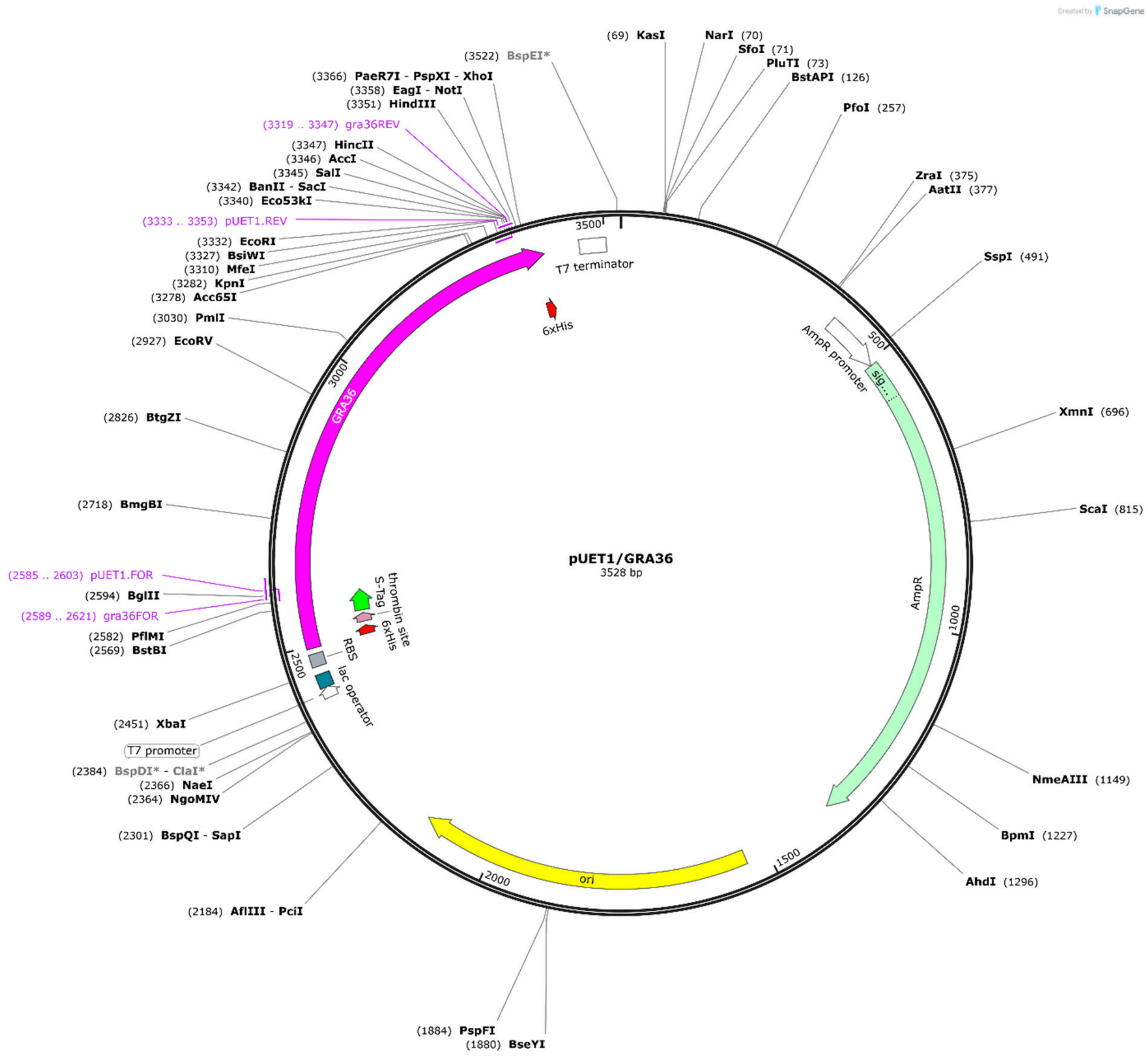

Figure S3. pUET1/GRA36 map.

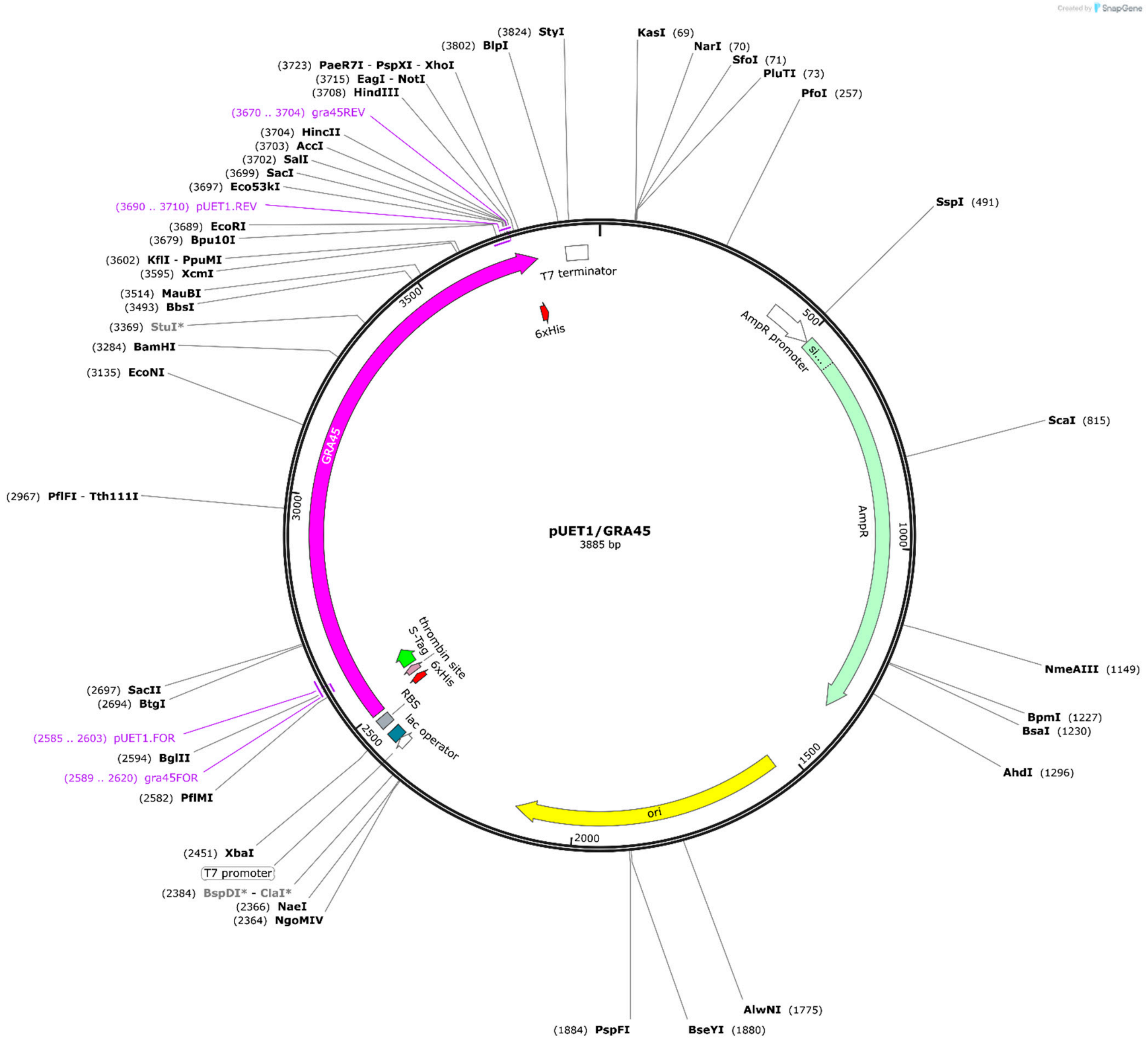

Figure S4. pUET1/GRA45 map.

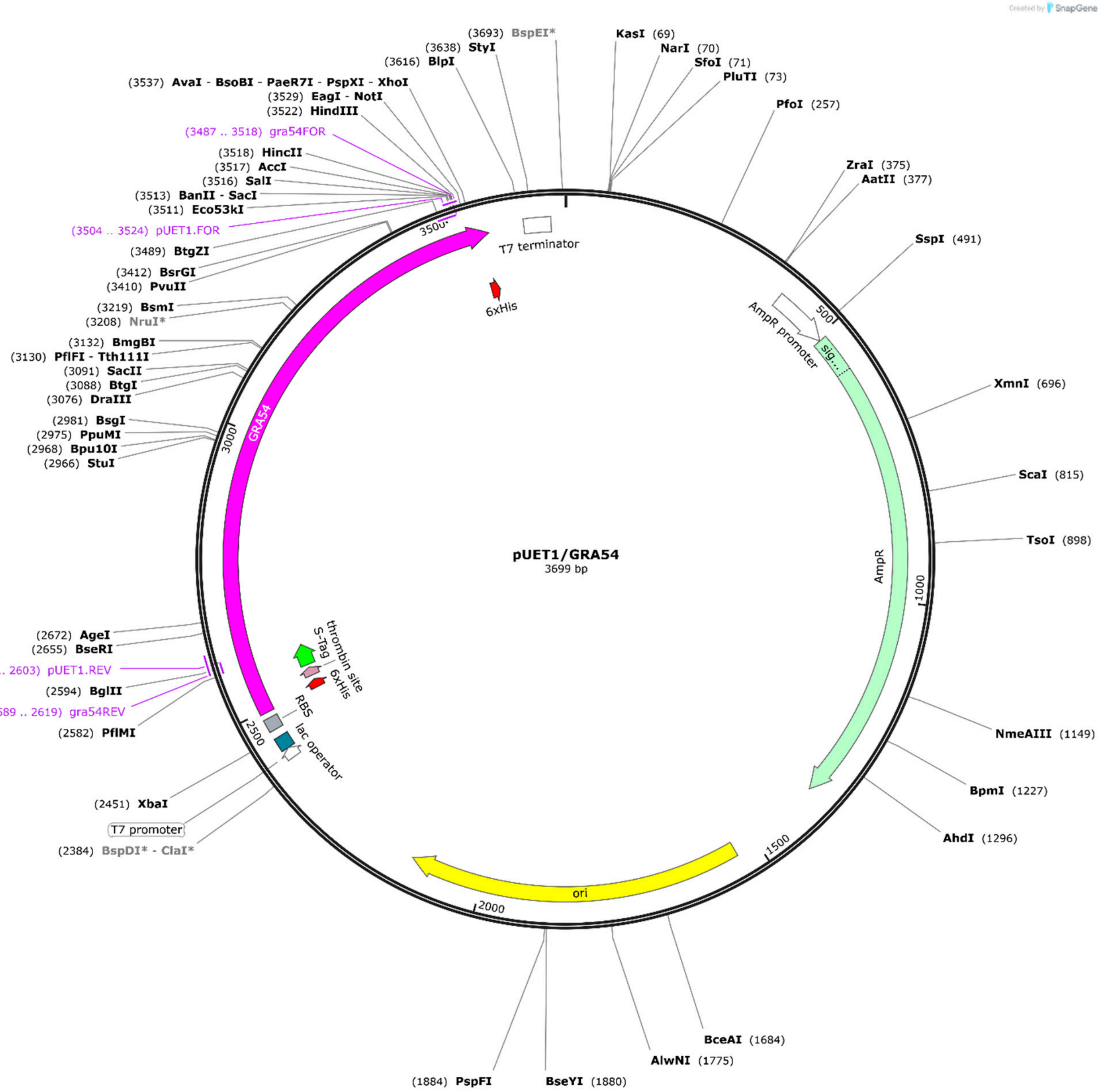

Figure S5. pUET1/GRA54 map.

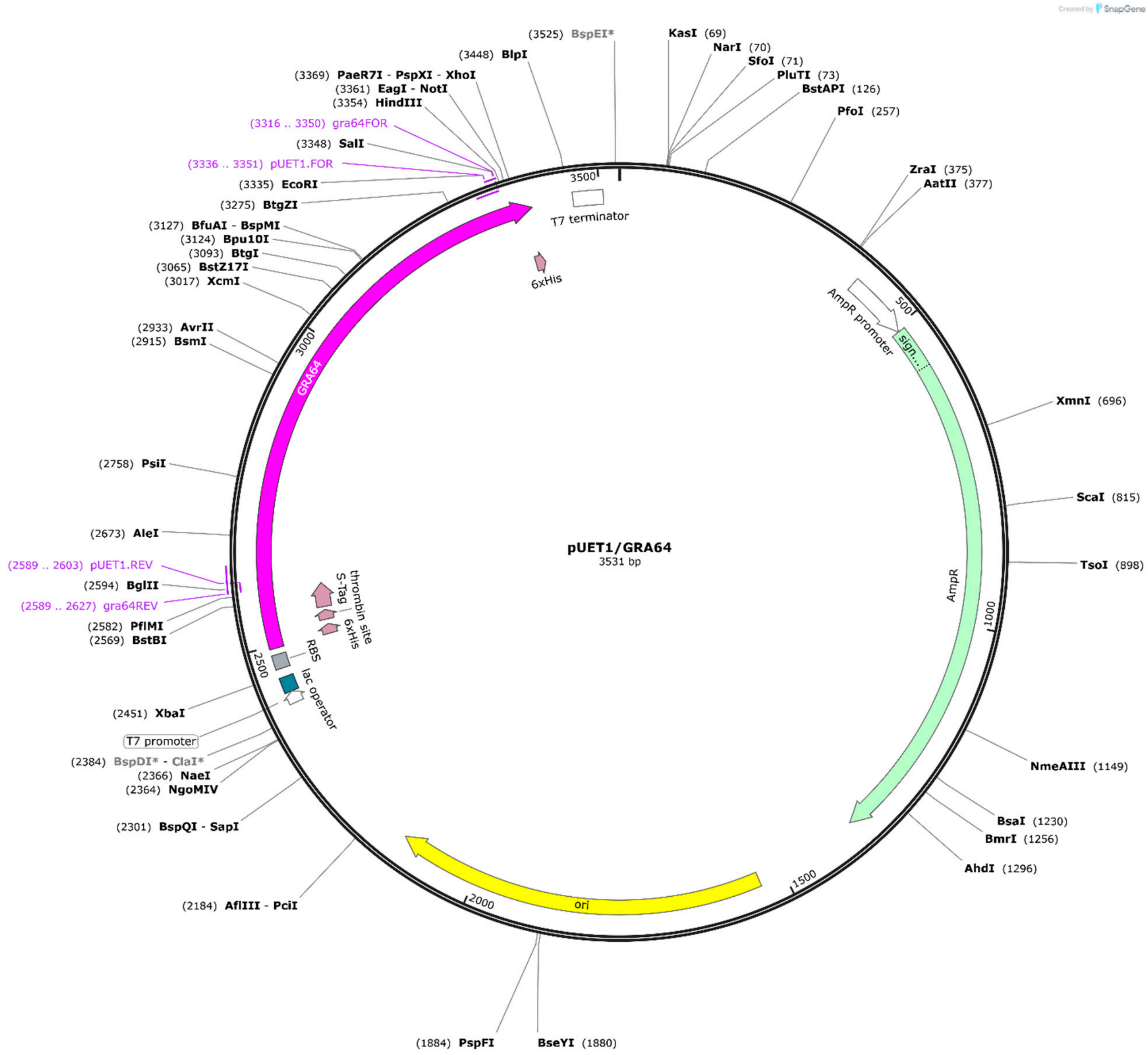

Figure S6. pUET1/GRA64 map.

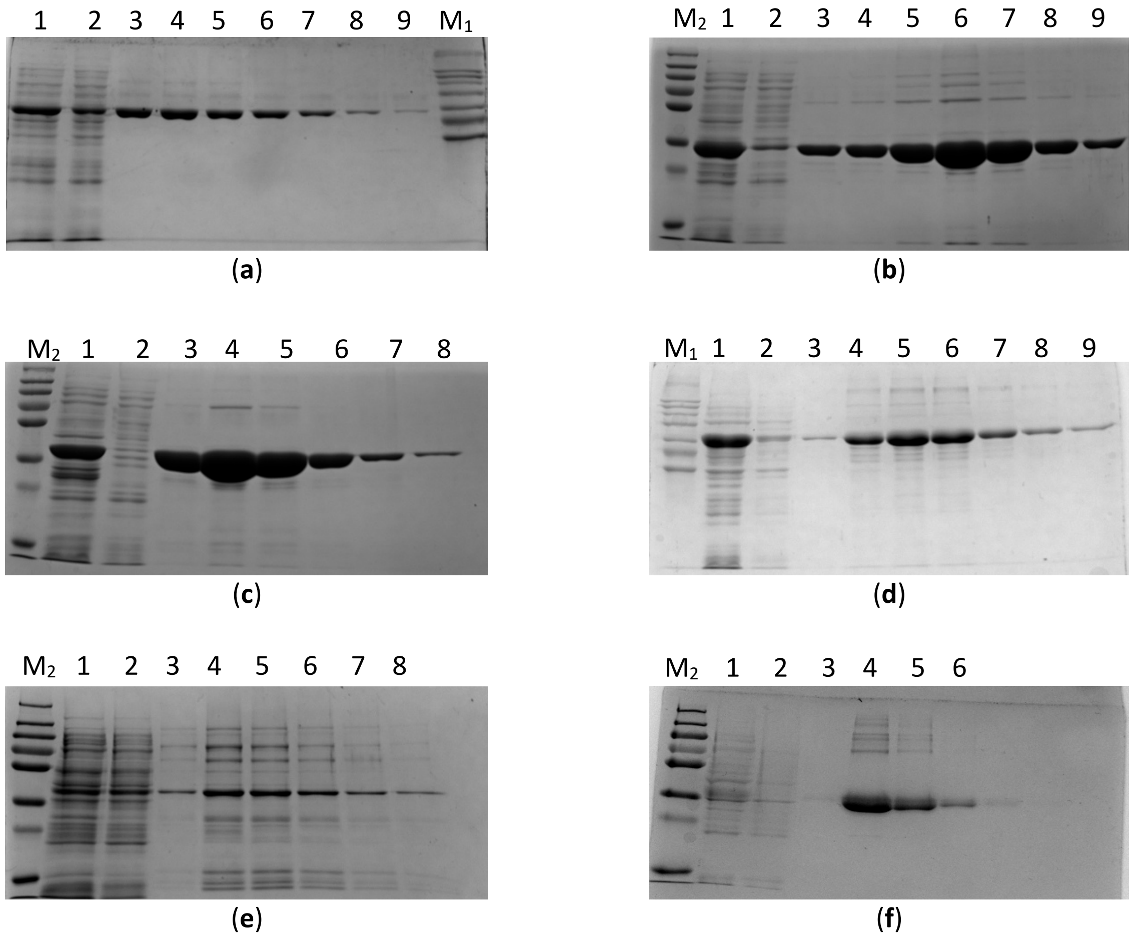

Figure S7. Images of 12% SDS–PAGE gels of purified protein preparations. **(a)** GRA29, **(b)** GRA35, **(c)** GRA36, **(d)** GRA45, **(e)** GRA54, **(f)** GRA64.

M_1—Molecular marker; SigmaMarker—high range (Sigma, catalogue number: S8320); M_2—Molecular marker PageRuler Plus Prestained Protein Ladder (Thermo Scientific, catalogue number: 26619); 1—Whole cell lysate; 2—Eluate; 3-9—Elution fractions.
